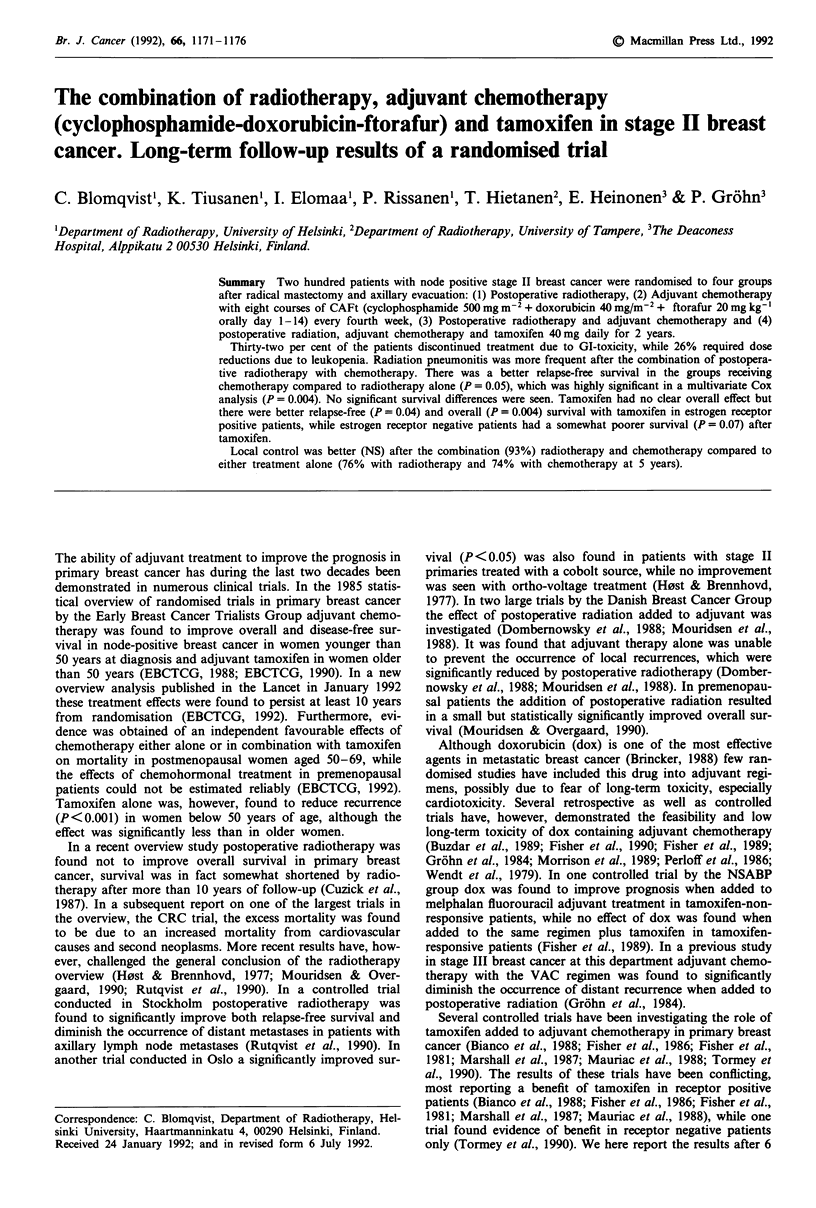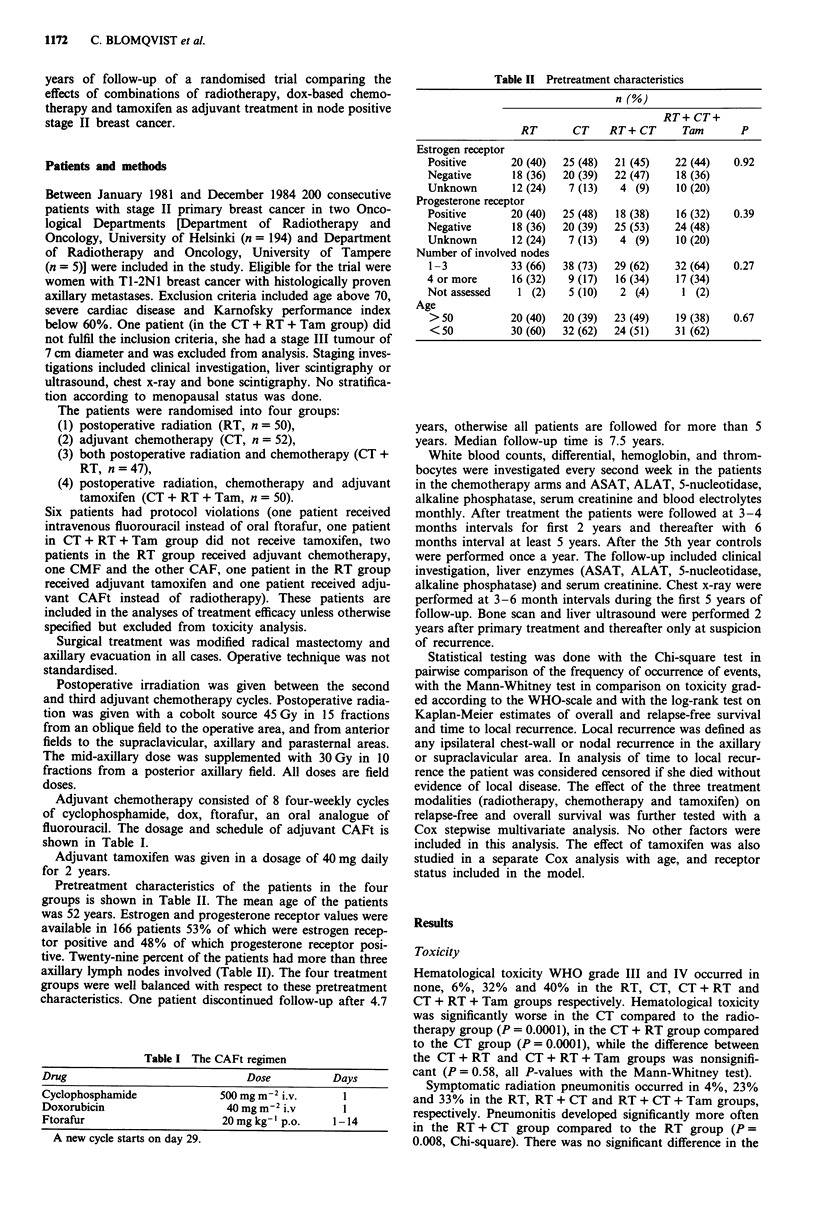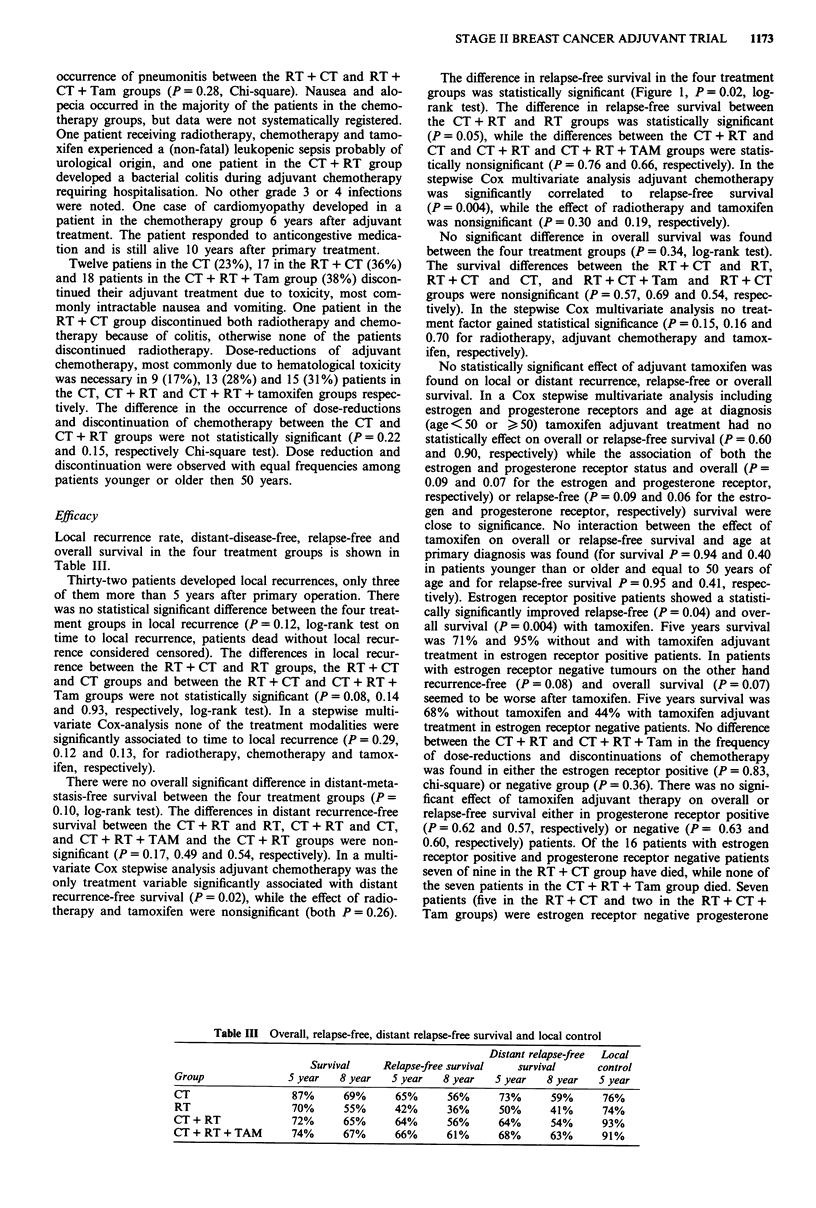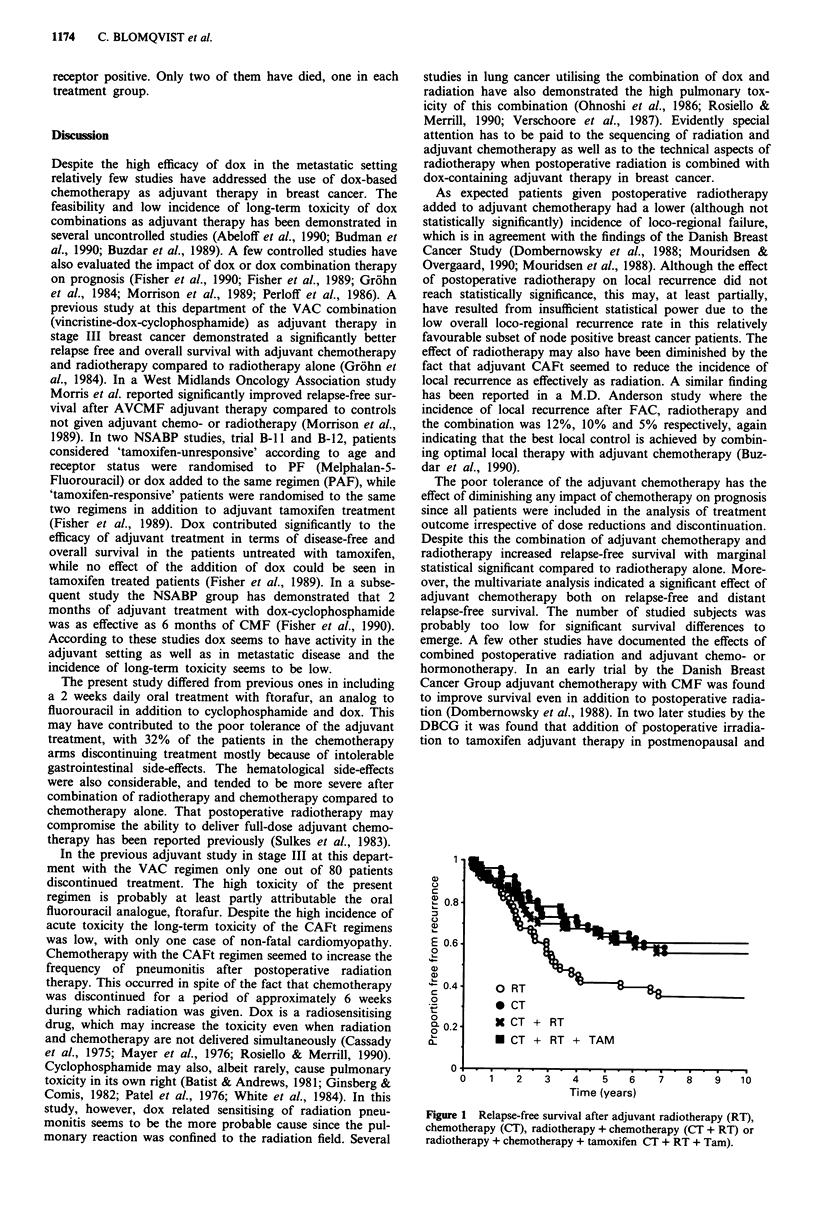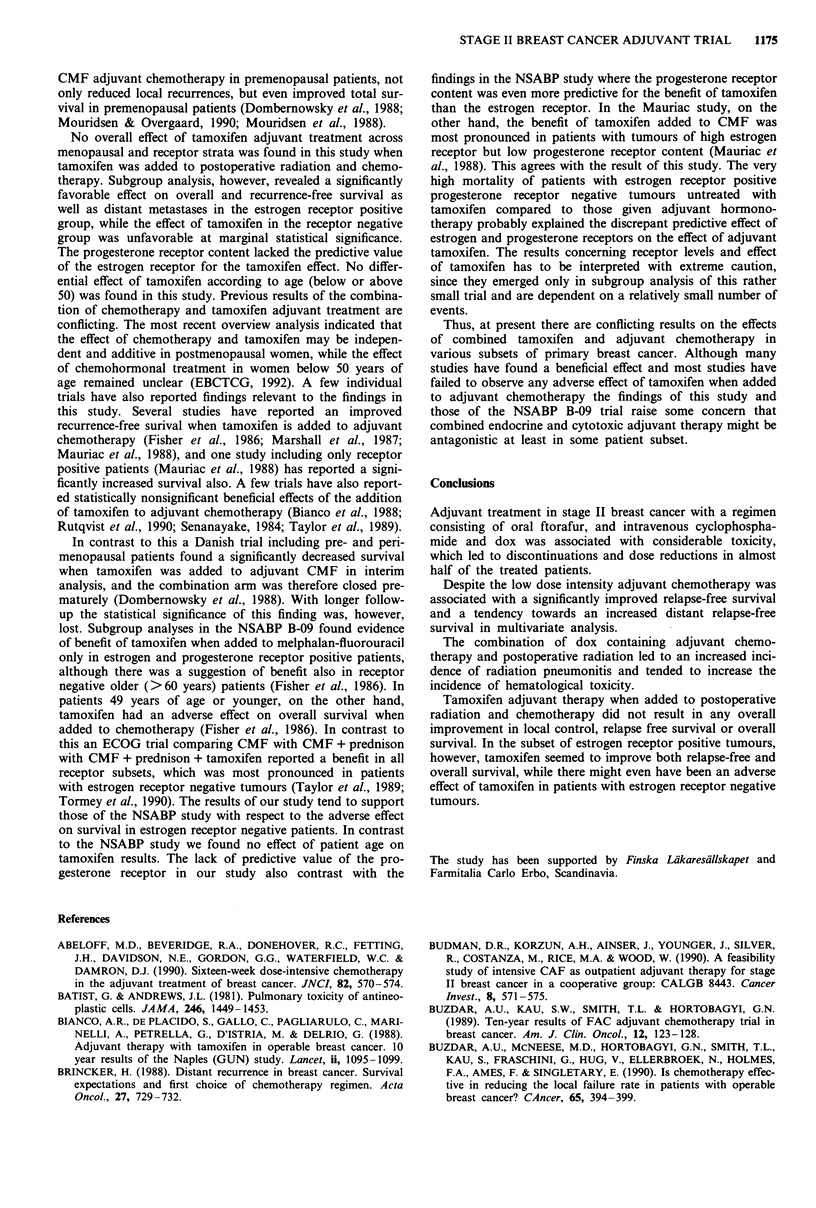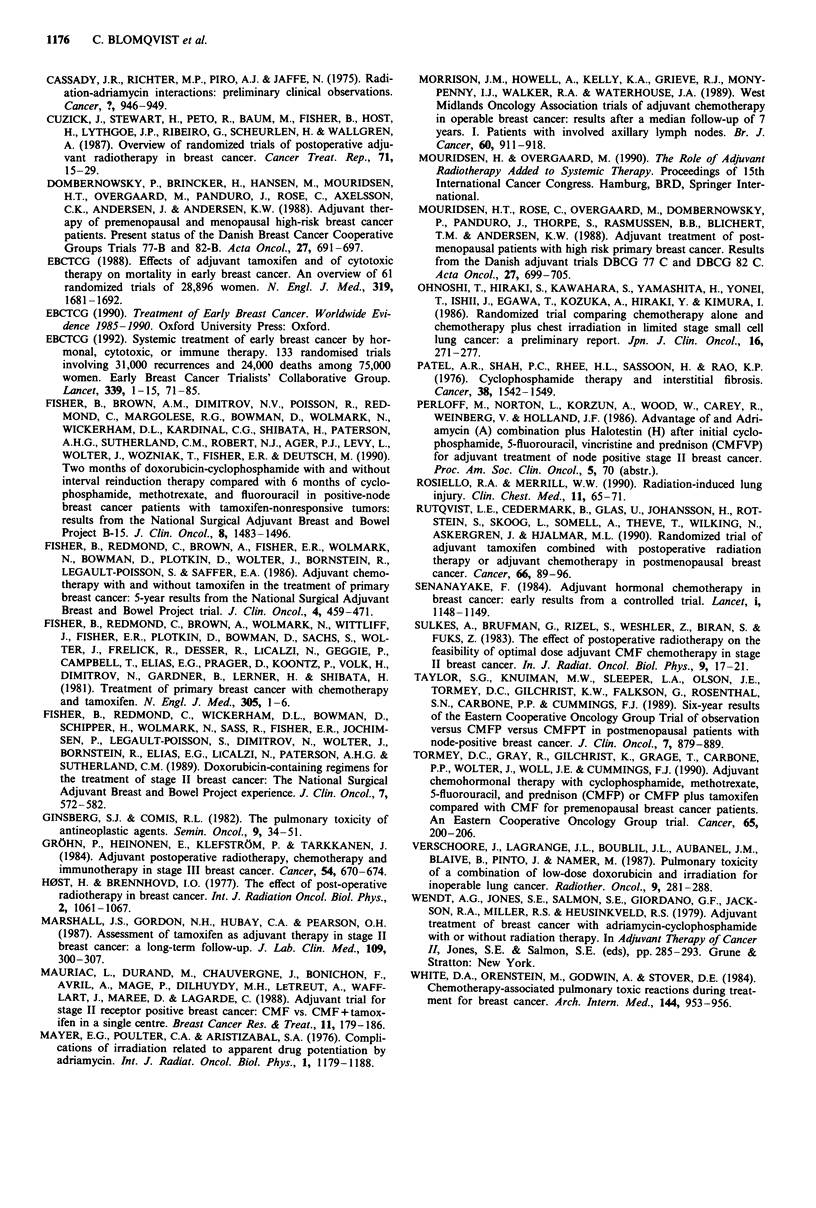# The combination of radiotherapy, adjuvant chemotherapy (cyclophosphamide-doxorubicin-ftorafur) and tamoxifen in stage II breast cancer. Long-term follow-up results of a randomised trial.

**DOI:** 10.1038/bjc.1992.430

**Published:** 1992-12

**Authors:** C. Blomqvist, K. Tiusanen, I. Elomaa, P. Rissanen, T. Hietanen, E. Heinonen, P. Gröhn

**Affiliations:** Department of Radiotherapy, University of Helsinki, Finland.

## Abstract

Two hundred patients with node positive stage II breast cancer were randomised to four groups after radical mastectomy and axillary evacuation: (1) Postoperative radiotherapy, (2) Adjuvant chemotherapy with eight courses of CAFt (cyclophosphamide 500 mg m-2 + doxorubicin 40 mg/m-2 + ftorafur 20 mg kg-1 orally day 1-14) every fourth week, (3) Postoperative radiotherapy and adjuvant chemotherapy and (4) postoperative radiation, adjuvant chemotherapy and tamoxifen 40 mg daily for 2 years. Thirty-two per cent of the patients discontinued treatment due to GI-toxicity, while 26% required dose reductions due to leukopenia. Radiation pneumonitis was more frequent after the combination of postoperative radiotherapy with chemotherapy. There was a better relapse-free survival in the groups receiving chemotherapy compared to radiotherapy alone (P = 0.05), which was highly significant in a multivariate Cox analysis (P = 0.004). No significant survival differences were seen. Tamoxifen had no clear overall effect but there were better relapse-free (P = 0.04) and overall (P = 0.004) survival with tamoxifen in estrogen receptor positive patients, while estrogen receptor negative patients had a somewhat poorer survival (P = 0.07) after tamoxifen. Local control was better (NS) after the combination (93%) radiotherapy and chemotherapy compared to either treatment alone (76% with radiotherapy and 74% with chemotherapy at 5 years).


Br. J. Cancer (1992), 66, 1171–1176 © Macmillan Press Ltd., 1992

### The combination of radiotherapy, adjuvant chemotherapy (cyclophosphamide-doxorubicin-ftorafur) and tamoxifen in stage II breast cancer. Long-term follow-up results of a randomised trial

C. Blomqvist[1], K. Tiusanen[1], I. Elomaa[1], P. Rissanen[1], T. Hietanen[2], E. Heinonen[3] & P. Gröhn[3]

[1]*Department of Radiotherapy, University of Helsinki,* [2]*Department of Radiotherapy, University of Tampere,* [3]*The Deaconess Hospital, Alppikatu 2 00530 Helsinki, Finland.*

**Summary** Two hundred patients with node positive stage II breast cancer were randomised to four groups after radical mastectomy and axillary evacuation: (1) Postoperative radiotherapy, (2) Adjuvant chemotherapy with eight courses of CAFt (cyclophosphamide 500 mg $m^{-2}$ + doxorubicin 40 mg/$m^{-2}$ + ftorafur 20 mg $kg^{-1}$ orally day 1–14) every fourth week, (3) Postoperative radiotherapy and adjuvant chemotherapy and (4) postoperative radiation, adjuvant chemotherapy and tamoxifen 40 mg daily for 2 years.

Thirty-two per cent of the patients discontinued treatment due to GI-toxicity, while 26% required dose reductions due to leukopenia. Radiation pneumonitis was more frequent after the combination of postoperative radiotherapy with chemotherapy. There was a better relapse-free survival in the groups receiving chemotherapy compared to radiotherapy alone ($P = 0.05$), which was highly significant in a multivariate Cox analysis ($P = 0.004$). No significant survival differences were seen. Tamoxifen had no clear overall effect but there were better relapse-free ($P = 0.04$) and overall ($P = 0.004$) survival with tamoxifen in estrogen receptor positive patients, while estrogen receptor negative patients had a somewhat poorer survival ($P = 0.07$) after tamoxifen.

Local control was better (NS) after the combination (93%) radiotherapy and chemotherapy compared to either treatment alone (76% with radiotherapy and 74% with chemotherapy at 5 years).

The ability of adjuvant treatment to improve the prognosis in primary breast cancer has during the last two decades been demonstrated in numerous clinical trials. In the 1985 statistical overview of randomised trials in primary breast cancer by the Early Breast Cancer Trialists Group adjuvant chemotherapy was found to improve overall and disease-free survival in node-positive breast cancer in women younger than 50 years at diagnosis and adjuvant tamoxifen in women older than 50 years (EBCTCG, 1988; EBCTCG, 1990). In a new overview analysis published in the Lancet in January 1992 these treatment effects were found to persist at least 10 years from randomisation (EBCTCG, 1992). Furthermore, evidence was obtained of an independent favourable effects of chemotherapy either alone or in combination with tamoxifen on mortality in postmenopausal women aged 50–69, while the effects of chemohormonal treatment in premenopausal patients could not be estimated reliably (EBCTCG, 1992). Tamoxifen alone was, however, found to reduce recurrence ($P < 0.001$) in women below 50 years of age, although the effect was significantly less than in older women.

In a recent overview study postoperative radiotherapy was found not to improve overall survival in primary breast cancer, survival was in fact somewhat shortened by radiotherapy after more than 10 years of follow-up (Cuzick *et al.*, 1987). In a subsequent report on one of the largest trials in the overview, the CRC trial, the excess mortality was found to be due to an increased mortality from cardiovascular causes and second neoplasms. More recent results have, however, challenged the general conclusion of the radiotherapy overview (Høst & Brennhovd, 1977; Mouridsen & Overgaard, 1990; Rutqvist *et al.*, 1990). In a controlled trial conducted in Stockholm postoperative radiotherapy was found to significantly improve both relapse-free survival and diminish the occurrence of distant metastases in patients with axillary lymph node metastases (Rutqvist *et al.*, 1990). In another trial conducted in Oslo a significantly improved survival ($P < 0.05$) was also found in patients with stage II primaries treated with a cobolt source, while no improvement was seen with ortho-voltage treatment (Høst & Brennhovd, 1977). In two large trials by the Danish Breast Cancer Group the effect of postoperative radiation added to adjuvant was investigated (Dombernowsky *et al.*, 1988; Mouridsen *et al.*, 1988). It was found that adjuvant therapy alone was unable to prevent the occurrence of local recurrences, which were significantly reduced by postoperative radiotherapy (Dombernowsky *et al.*, 1988; Mouridsen *et al.*, 1988). In premenopausal patients the addition of postoperative radiation resulted in a small but statistically significantly improved overall survival (Mouridsen & Overgaard, 1990).

Although doxorubicin (dox) is one of the most effective agents in metastatic breast cancer (Brincker, 1988) few randomised studies have included this drug into adjuvant regimens, possibly due to fear of long-term toxicity, especially cardiotoxicity. Several retrospective as well as controlled trials have, however, demonstrated the feasibility and low long-term toxicity of dox containing adjuvant chemotherapy (Buzdar *et al.*, 1989; Fisher *et al.*, 1990; Fisher *et al.*, 1989; Gröhn *et al.*, 1984; Morrison *et al.*, 1989; Perloff *et al.*, 1986; Wendt *et al.*, 1979). In one controlled trial by the NSABP group dox was found to improve prognosis when added to melphalan fluorouracil adjuvant treatment in tamoxifen-non-responsive patients, while no effect of dox was found when added to the same regimen plus tamoxifen in tamoxifen-responsive patients (Fisher *et al.*, 1989). In a previous study in stage III breast cancer at this department adjuvant chemotherapy with the VAC regimen was found to significantly diminish the occurrence of distant recurrence when added to postoperative radiation (Gröhn *et al.*, 1984).

Several controlled trials have been investigating the role of tamoxifen added to adjuvant chemotherapy in primary breast cancer (Bianco *et al.*, 1988; Fisher *et al.*, 1986; Fisher *et al.*, 1981; Marshall *et al.*, 1987; Mauriac *et al.*, 1988; Tormey *et al.*, 1990). The results of these trials have been conflicting, most reporting a benefit of tamoxifen in receptor positive patients (Bianco *et al.*, 1988; Fisher *et al.*, 1986; Fisher *et al.*, 1981; Marshall *et al.*, 1987; Mauriac *et al.*, 1988), while one trial found evidence of benefit in receptor negative patients only (Tormey *et al.*, 1990). We here report the results after 6

Correspondence: C. Blomqvist, Department of Radiotherapy, Helsinki University, Haartmanninkatu 4, 00290 Helsinki, Finland.
Received 24 January 1992; and in revised form 6 July 1992.

years of follow-up of a randomised trial comparing the effects of combinations of radiotherapy, dox-based chemotherapy and tamoxifen as adjuvant treatment in node positive stage II breast cancer.

### Patients and methods

Between January 1981 and December 1984 200 consecutive patients with stage II primary breast cancer in two Oncological Departments [Department of Radiotherapy and Oncology, University of Helsinki ($n = 194$) and Department of Radiotherapy and Oncology, University of Tampere ($n = 5$)] were included in the study. Eligible for the trial were women with T1-2N1 breast cancer with histologically proven axillary metastases. Exclusion criteria included age above 70, severe cardiac disease and Karnofsky performance index below 60%. One patient (in the CT + RT + Tam group) did not fulfil the inclusion criteria, she had a stage III tumour of 7 cm diameter and was excluded from analysis. Staging investigations included clinical investigation, liver scintigraphy or ultrasound, chest x-ray and bone scintigraphy. No stratification according to menopausal status was done.

The patients were randomised into four groups:
(1) postoperative radiation (RT, $n = 50$),
(2) adjuvant chemotherapy (CT, $n = 52$),
(3) both postoperative radiation and chemotherapy (CT + RT, $n = 47$),
(4) postoperative radiation, chemotherapy and adjuvant tamoxifen (CT + RT + Tam, $n = 50$).

Six patients had protocol violations (one patient received intravenous fluorouracil instead of oral ftorafur, one patient in CT + RT + Tam group did not receive tamoxifen, two patients in the RT group received adjuvant chemotherapy, one CMF and the other CAF, one patient in the RT group received adjuvant tamoxifen and one patient received adjuvant CAFt instead of radiotherapy). These patients are included in the analyses of treatment efficacy unless otherwise specified but excluded from toxicity analysis.

Surgical treatment was modified radical mastectomy and axillary evacuation in all cases. Operative technique was not standardised.

Postoperative irradiation was given between the second and third adjuvant chemotherapy cycles. Postoperative radiation was given with a cobolt source 45 Gy in 15 fractions from an oblique field to the operative area, and from anterior fields to the supraclavicular, axillary and parasternal areas. The mid-axillary dose was supplemented with 30 Gy in 10 fractions from a posterior axillary field. All doses are field doses.

Adjuvant chemotherapy consisted of 8 four-weekly cycles of cyclophosphamide, dox, ftorafur, an oral analogue of fluorouracil. The dosage and schedule of adjuvant CAFt is shown in Table I.

Adjuvant tamoxifen was given in a dosage of 40 mg daily for 2 years.

Pretreatment characteristics of the patients in the four groups is shown in Table II. The mean age of the patients was 52 years. Estrogen and progesterone receptor values were available in 166 patients 53% of which were estrogen receptor positive and 48% of which progesterone receptor positive. Twenty-nine percent of the patients had more than three axillary lymph nodes involved (Table II). The four treatment groups were well balanced with respect to these pretreatment characteristics. One patient discontinued follow-up after 4.7 years, otherwise all patients are followed for more than 5 years. Median follow-up time is 7.5 years.

**Table I** The CAFt regimen

| Drug | Dose | Days |
|---|---|---|
| Cyclophosphamide | 500 mg $m^{-2}$ i.v. | 1 |
| Doxorubicin | 40 mg $m^{-2}$ i.v | 1 |
| Ftorafur | 20 mg $kg^{-1}$ p.o. | 1–14 |

A new cycle starts on day 29.

**Table II** Pretreatment characteristics

| | n (%) | | | | |
|---|---|---|---|---|---|
| | RT | CT | RT + CT | RT + CT + Tam | P |
| Estrogen receptor | | | | | |
| Positive | 20 (40) | 25 (48) | 21 (45) | 22 (44) | 0.92 |
| Negative | 18 (36) | 20 (39) | 22 (47) | 18 (36) | |
| Unknown | 12 (24) | 7 (13) | 4 (9) | 10 (20) | |
| Progesterone receptor | | | | | |
| Positive | 20 (40) | 25 (48) | 18 (38) | 16 (32) | 0.39 |
| Negative | 18 (36) | 20 (39) | 25 (53) | 24 (48) | |
| Unknown | 12 (24) | 7 (13) | 4 (9) | 10 (20) | |
| Number of involved nodes | | | | | |
| 1–3 | 33 (66) | 38 (73) | 29 (62) | 32 (64) | 0.27 |
| 4 or more | 16 (32) | 9 (17) | 16 (34) | 17 (34) | |
| Not assessed | 1 (2) | 5 (10) | 2 (4) | 1 (2) | |
| Age | | | | | |
| >50 | 20 (40) | 20 (39) | 23 (49) | 19 (38) | 0.67 |
| <50 | 30 (60) | 32 (62) | 24 (51) | 31 (62) | |

White blood counts, differential, hemoglobin, and thrombocytes were investigated every second week in the patients in the chemotherapy arms and ASAT, ALAT, 5-nucleotidase, alkaline phosphatase, serum creatinine and blood electrolytes monthly. After treatment the patients were followed at 3–4 months intervals for first 2 years and thereafter with 6 months interval at least 5 years. After the 5th year controls were performed once a year. The follow-up included clinical investigation, liver enzymes (ASAT, ALAT, 5-nucleotidase, alkaline phosphatase) and serum creatinine. Chest x-ray were performed at 3–6 month intervals during the first 5 years of follow-up. Bone scan and liver ultrasound were performed 2 years after primary treatment and thereafter only at suspicion of recurrence.

Statistical testing was done with the Chi-square test in pairwise comparison of the frequency of occurrence of events, with the Mann-Whitney test in comparison on toxicity graded according to the WHO-scale and with the log-rank test on Kaplan-Meier estimates of overall and relapse-free survival and time to local recurrence. Local recurrence was defined as any ipsilateral chest-wall or nodal recurrence in the axillary or supraclavicular area. In analysis of time to local recurrence the patient was considered censored if she died without evidence of local disease. The effect of the three treatment modalities (radiotherapy, chemotherapy and tamoxifen) on relapse-free and overall survival was further tested with a Cox stepwise multivariate analysis. No other factors were included in this analysis. The effect of tamoxifen was also studied in a separate Cox analysis with age, and receptor status included in the model.

### Results

### Toxicity

Hematological toxicity WHO grade III and IV occurred in none, 6%, 32% and 40% in the RT, CT, CT + RT and CT + RT + Tam groups respectively. Hematological toxicity was significantly worse in the CT compared to the radiotherapy group ($P = 0.0001$), in the CT + RT group compared to the CT group ($P = 0.0001$), while the difference between the CT + RT and CT + RT + Tam groups was nonsignificant ($P = 0.58$, all $P$-values with the Mann-Whitney test).

Symptomatic radiation pneumonitis occurred in 4%, 23% and 33% in the RT, RT + CT and RT + CT + Tam groups, respectively. Pneumonitis developed significantly more often in the RT + CT group compared to the RT group ($P = 0.008$, Chi-square). There was no significant difference in the occurrence of pneumonitis between the RT + CT and RT + CT + Tam groups ($P = 0.28$, Chi-square). Nausea and alopecia occurred in the majority of the patients in the chemotherapy groups, but data were not systematically registered. One patient receiving radiotherapy, chemotherapy and tamoxifen experienced a (non-fatal) leukopenic sepsis probably of urological origin, and one patient in the CT + RT group developed a bacterial colitis during adjuvant chemotherapy requiring hospitalisation. No other grade 3 or 4 infections were noted. One case of cardiomyopathy developed in a patient in the chemotherapy group 6 years after adjuvant treatment. The patient responded to anticongestive medication and is still alive 10 years after primary treatment.

Twelve patiens in the CT (23%), 17 in the RT + CT (36%) and 18 patients in the CT + RT + Tam group (38%) discontinued their adjuvant treatment due to toxicity, most commonly intractable nausea and vomiting. One patient in the RT + CT group discontinued both radiotherapy and chemotherapy because of colitis, otherwise none of the patients discontinued radiotherapy. Dose-reductions of adjuvant chemotherapy, most commonly due to hematological toxicity was necessary in 9 (17%), 13 (28%) and 15 (31%) patients in the CT, CT + RT and CT + RT + tamoxifen groups respectively. The difference in the occurrence of dose-reductions and discontinuation of chemotherapy between the CT and CT + RT groups were not statistically significant ($P = 0.22$ and 0.15, respectively Chi-square test). Dose reduction and discontinuation were observed with equal frequencies among patients younger or older then 50 years.

### *Efficacy*

Local recurrence rate, distant-disease-free, relapse-free and overall survival in the four treatment groups is shown in Table III.

Thirty-two patients developed local recurrences, only three of them more than 5 years after primary operation. There was no statistical significant difference between the four treatment groups in local recurrence ($P = 0.12$, log-rank test on time to local recurrence, patients dead without local recurrence considered censored). The differences in local recurrence between the RT + CT and RT groups, the RT + CT and CT groups and between the RT + CT and CT + RT + Tam groups were not statistically significant ($P = 0.08$, 0.14 and 0.93, respectively, log-rank test). In a stepwise multivariate Cox-analysis none of the treatment modalities were significantly associated to time to local recurrence ($P = 0.29$, 0.12 and 0.13, for radiotherapy, chemotherapy and tamoxifen, respectively).

There were no overall significant difference in distant-metastasis-free survival between the four treatment groups ($P = 0.10$, log-rank test). The differences in distant recurrence-free survival between the CT + RT and RT, CT + RT and CT, and CT + RT + TAM and the CT + RT groups were non-significant ($P = 0.17$, 0.49 and 0.54, respectively). In a multivariate Cox stepwise analysis adjuvant chemotherapy was the only treatment variable significantly associated with distant recurrence-free survival ($P = 0.02$), while the effect of radiotherapy and tamoxifen were nonsignificant (both $P = 0.26$).

The difference in relapse-free survival in the four treatment groups was statistically significant (Figure 1, $P = 0.02$, log-rank test). The difference in relapse-free survival between the CT + RT and RT groups was statistically significant ($P = 0.05$), while the differences between the CT + RT and CT and CT + RT and CT + RT + TAM groups were statistically nonsignificant ($P = 0.76$ and 0.66, respectively). In the stepwise Cox multivariate analysis adjuvant chemotherapy was significantly correlated to relapse-free survival ($P = 0.004$), while the effect of radiotherapy and tamoxifen was nonsignificant ($P = 0.30$ and 0.19, respectively).

No significant difference in overall survival was found between the four treatment groups ($P = 0.34$, log-rank test). The survival differences between the RT + CT and RT, RT + CT and CT, and RT + CT + Tam and RT + CT groups were nonsignificant ($P = 0.57$, 0.69 and 0.54, respectively). In the stepwise Cox multivariate analysis no treatment factor gained statistical significance ($P = 0.15$, 0.16 and 0.70 for radiotherapy, adjuvant chemotherapy and tamoxifen, respectively).

No statistically significant effect of adjuvant tamoxifen was found on local or distant recurrence, relapse-free or overall survival. In a Cox stepwise multivariate analysis including estrogen and progesterone receptors and age at diagnosis (age $< 50$ or $\geqslant 50$) tamoxifen adjuvant treatment had no statistically effect on overall or relapse-free survival ($P = 0.60$ and 0.90, respectively) while the association of both the estrogen and progesterone receptor status and overall ($P = 0.09$ and 0.07 for the estrogen and progesterone receptor, respectively) or relapse-free ($P = 0.09$ and 0.06 for the estrogen and progesterone receptor, respectively) survival were close to significance. No interaction between the effect of tamoxifen on overall or relapse-free survival and age at primary diagnosis was found (for survival $P = 0.94$ and 0.40 in patients younger than or older and equal to 50 years of age and for relapse-free survival $P = 0.95$ and 0.41, respectively). Estrogen receptor positive patients showed a statistically significantly improved relapse-free ($P = 0.04$) and overall survival ($P = 0.004$) with tamoxifen. Five years survival was 71% and 95% without and with tamoxifen adjuvant treatment in estrogen receptor positive patients. In patients with estrogen receptor negative tumours on the other hand recurrence-free ($P = 0.08$) and overall survival ($P = 0.07$) seemed to be worse after tamoxifen. Five years survival was 68% without tamoxifen and 44% with tamoxifen adjuvant treatment in estrogen receptor negative patients. No difference between the CT + RT and CT + RT + Tam in the frequency of dose-reductions and discontinuations of chemotherapy was found in either the estrogen receptor positive ($P = 0.83$, chi-square) or negative group ($P = 0.36$). There was no significant effect of tamoxifen adjuvant therapy on overall or relapse-free survival either in progesterone receptor positive ($P = 0.62$ and 0.57, respectively) or negative ($P = 0.63$ and 0.60, respectively) patients. Of the 16 patients with estrogen receptor positive and progesterone receptor negative patients seven of nine in the RT + CT group have died, while none of the seven patients in the CT + RT + Tam group died. Seven patients (five in the RT + CT and two in the RT + CT + Tam groups) were estrogen receptor negative progesterone

**Table III** Overall, relapse-free, distant relapse-free survival and local control

| Group | *Survival* 5 year | *Survival* 8 year | *Relapse-free survival* 5 year | *Relapse-free survival* 8 year | *Distant relapse-free survival* 5 year | *Distant relapse-free survival* 8 year | *Local control* 5 year |
|---|---|---|---|---|---|---|---|
| CT | 87% | 69% | 65% | 56% | 73% | 59% | 76% |
| RT | 70% | 55% | 42% | 36% | 50% | 41% | 74% |
| CT + RT | 72% | 65% | 64% | 56% | 64% | 54% | 93% |
| CT + RT + TAM | 74% | 67% | 66% | 61% | 68% | 63% | 91% |

receptor positive. Only two of them have died, one in each treatment group.

### Discussion

Despite the high efficacy of dox in the metastatic setting relatively few studies have addressed the use of dox-based chemotherapy as adjuvant therapy in breast cancer. The feasibility and low incidence of long-term toxicity of dox combinations as adjuvant therapy has been demonstrated in several uncontrolled studies (Abeloff et al., 1990; Budman et al., 1990; Buzdar et al., 1989). A few controlled studies have also evaluated the impact of dox or dox combination therapy on prognosis (Fisher et al., 1990; Fisher et al., 1989; Gröhn et al., 1984; Morrison et al., 1989; Perloff et al., 1986). A previous study at this department of the VAC combination (vincristine-dox-cyclophosphamide) as adjuvant therapy in stage III breast cancer demonstrated a significantly better relapse free and overall survival with adjuvant chemotherapy and radiotherapy compared to radiotherapy alone (Gröhn et al., 1984). In a West Midlands Oncology Association study Morris et al. reported significantly improved relapse-free survival after AVCMF adjuvant therapy compared to controls not given adjuvant chemo- or radiotherapy (Morrison et al., 1989). In two NSABP studies, trial B-11 and B-12, patients considered 'tamoxifen-unresponsive' according to age and receptor status were randomised to PF (Melphalan-5-Fluorouracil) or dox added to the same regimen (PAF), while 'tamoxifen-responsive' patients were randomised to the same two regimens in addition to adjuvant tamoxifen treatment (Fisher et al., 1989). Dox contributed significantly to the efficacy of adjuvant treatment in terms of disease-free and overall survival in the patients untreated with tamoxifen, while no effect of the addition of dox could be seen in tamoxifen treated patients (Fisher et al., 1989). In a subsequent study the NSABP group has demonstrated that 2 months of adjuvant treatment with dox-cyclophosphamide was as effective as 6 months of CMF (Fisher et al., 1990). According to these studies dox seems to have activity in the adjuvant setting as well as in metastatic disease and the incidence of long-term toxicity seems to be low.

The present study differed from previous ones in including a 2 weeks daily oral treatment with ftorafur, an analog to fluorouracil in addition to cyclophosphamide and dox. This may have contributed to the poor tolerance of the adjuvant treatment, with 32% of the patients in the chemotherapy arms discontinuing treatment mostly because of intolerable gastrointestinal side-effects. The hematological side-effects were also considerable, and tended to be more severe after combination of radiotherapy and chemotherapy compared to chemotherapy alone. That postoperative radiotherapy may compromise the ability to deliver full-dose adjuvant chemotherapy has been reported previously (Sulkes et al., 1983).

In the previous adjuvant study in stage III at this department with the VAC regimen only one out of 80 patients discontinued treatment. The high toxicity of the present regimen is probably at least partly attributable the oral fluorouracil analogue, ftorafur. Despite the high incidence of acute toxicity the long-term toxicity of the CAFt regimens was low, with only one case of non-fatal cardiomyopathy. Chemotherapy with the CAFt regimen seemed to increase the frequency of pneumonitis after postoperative radiation therapy. This occurred in spite of the fact that chemotherapy was discontinued for a period of approximately 6 weeks during which radiation was given. Dox is a radiosensitising drug, which may increase the toxicity even when radiation and chemotherapy are not delivered simultaneously (Cassady et al., 1975; Mayer et al., 1976; Rosiello & Merrill, 1990). Cyclophosphamide may also, albeit rarely, cause pulmonary toxicity in its own right (Batist & Andrews, 1981; Ginsberg & Comis, 1982; Patel et al., 1976; White et al., 1984). In this study, however, dox related sensitising of radiation pneumonitis seems to be the more probable cause since the pulmonary reaction was confined to the radiation field. Several studies in lung cancer utilising the combination of dox and radiation have also demonstrated the high pulmonary toxicity of this combination (Ohnoshi et al., 1986; Rosiello & Merrill, 1990; Verschoore et al., 1987). Evidently special attention has to be paid to the sequencing of radiation and adjuvant chemotherapy as well as to the technical aspects of radiotherapy when postoperative radiation is combined with dox-containing adjuvant therapy in breast cancer.

As expected patients given postoperative radiotherapy added to adjuvant chemotherapy had a lower (although not statistically significantly) incidence of loco-regional failure, which is in agreement with the findings of the Danish Breast Cancer Study (Dombernowsky et al., 1988; Mouridsen & Overgaard, 1990; Mouridsen et al., 1988). Although the effect of postoperative radiotherapy on local recurrence did not reach statistically significance, this may, at least partially, have resulted from insufficient statistical power due to the low overall loco-regional recurrence rate in this relatively favourable subset of node positive breast cancer patients. The effect of radiotherapy may also have been diminished by the fact that adjuvant CAFt seemed to reduce the incidence of local recurrence as effectively as radiation. A similar finding has been reported in a M.D. Anderson study where the incidence of local recurrence after FAC, radiotherapy and the combination was 12%, 10% and 5% respectively, again indicating that the best local control is achieved by combining optimal local therapy with adjuvant chemotherapy (Buzdar et al., 1990).

The poor tolerance of the adjuvant chemotherapy has the effect of diminishing any impact of chemotherapy on prognosis since all patients were included in the analysis of treatment outcome irrespective of dose reductions and discontinuation. Despite this the combination of adjuvant chemotherapy and radiotherapy increased relapse-free survival with marginal statistical significant compared to radiotherapy alone. Moreover, the multivariate analysis indicated a significant effect of adjuvant chemotherapy both on relapse-free and distant relapse-free survival. The number of studied subjects was probably too low for significant survival differences to emerge. A few other studies have documented the effects of combined postoperative radiation and adjuvant chemo- or hormonotherapy. In an early trial by the Danish Breast Cancer Group adjuvant chemotherapy with CMF was found to improve survival even in addition to postoperative radiation (Dombernowsky et al., 1988). In two later studies by the DBCG it was found that addition of postoperative irradiation to tamoxifen adjuvant therapy in postmenopausal and

**Figure 1** Relapse-free survival after adjuvant radiotherapy (RT), chemotherapy (CT), radiotherapy + chemotherapy (CT + RT) or radiotherapy + chemotherapy + tamoxifen CT + RT + Tam).

CMF adjuvant chemotherapy in premenopausal patients, not only reduced local recurrences, but even improved total survival in premenopausal patients (Dombernowsky *et al.*, 1988; Mouridsen & Overgaard, 1990; Mouridsen *et al.*, 1988).

No overall effect of tamoxifen adjuvant treatment across menopausal and receptor strata was found in this study when tamoxifen was added to postoperative radiation and chemotherapy. Subgroup analysis, however, revealed a significantly favorable effect on overall and recurrence-free survival as well as distant metastases in the estrogen receptor positive group, while the effect of tamoxifen in the receptor negative group was unfavorable at marginal statistical significance. The progesterone receptor content lacked the predictive value of the estrogen receptor for the tamoxifen effect. No differential effect of tamoxifen according to age (below or above 50) was found in this study. Previous results of the combination of chemotherapy and tamoxifen adjuvant treatment are conflicting. The most recent overview analysis indicated that the effect of chemotherapy and tamoxifen may be independent and additive in postmenopausal women, while the effect of chemohormonal treatment in women below 50 years of age remained unclear (EBCTCG, 1992). A few individual trials have also reported findings relevant to the findings in this study. Several studies have reported an improved recurrence-free surival when tamoxifen is added to adjuvant chemotherapy (Fisher *et al.*, 1986; Marshall *et al.*, 1987; Mauriac *et al.*, 1988), and one study including only receptor positive patients (Mauriac *et al.*, 1988) has reported a significantly increased survival also. A few trials have also reported statistically nonsignificant beneficial effects of the addition of tamoxifen to adjuvant chemotherapy (Bianco *et al.*, 1988; Rutqvist *et al.*, 1990; Senanayake, 1984; Taylor *et al.*, 1989).

In contrast to this a Danish trial including pre- and perimenopausal patients found a significantly decreased survival when tamoxifen was added to adjuvant CMF in interim analysis, and the combination arm was therefore closed prematurely (Dombernowsky *et al.*, 1988). With longer follow-up the statistical significance of this finding was, however, lost. Subgroup analyses in the NSABP B-09 found evidence of benefit of tamoxifen when added to melphalan-fluorouracil only in estrogen and progesterone receptor positive patients, although there was a suggestion of benefit also in receptor negative older (>60 years) patients (Fisher *et al.*, 1986). In patients 49 years of age or younger, on the other hand, tamoxifen had an adverse effect on overall survival when added to chemotherapy (Fisher *et al.*, 1986). In contrast to this an ECOG trial comparing CMF with CMF + prednison with CMF + prednison + tamoxifen reported a benefit in all receptor subsets, which was most pronounced in patients with estrogen receptor negative tumours (Taylor *et al.*, 1989; Tormey *et al.*, 1990). The results of our study tend to support those of the NSABP study with respect to the adverse effect on survival in estrogen receptor negative patients. In contrast to the NSABP study we found no effect of patient age on tamoxifen results. The lack of predictive value of the progesterone receptor in our study also contrast with the findings in the NSABP study where the progesterone receptor content was even more predictive for the benefit of tamoxifen than the estrogen receptor. In the Mauriac study, on the other hand, the benefit of tamoxifen added to CMF was most pronounced in patients with tumours of high estrogen receptor but low progesterone receptor content (Mauriac *et al.*, 1988). This agrees with the result of this study. The very high mortality of patients with estrogen receptor positive progesterone receptor negative tumours untreated with tamoxifen compared to those given adjuvant hormonotherapy probably explained the discrepant predictive effect of estrogen and progesterone receptors on the effect of adjuvant tamoxifen. The results concerning receptor levels and effect of tamoxifen has to be interpreted with extreme caution, since they emerged only in subgroup analysis of this rather small trial and are dependent on a relatively small number of events.

Thus, at present there are conflicting results on the effects of combined tamoxifen and adjuvant chemotherapy in various subsets of primary breast cancer. Although many studies have found a beneficial effect and most studies have failed to observe any adverse effect of tamoxifen when added to adjuvant chemotherapy the findings of this study and those of the NSABP B-09 trial raise some concern that combined endocrine and cytotoxic adjuvant therapy might be antagonistic at least in some patient subset.

### Conclusions

Adjuvant treatment in stage II breast cancer with a regimen consisting of oral ftorafur, and intravenous cyclophosphamide and dox was associated with considerable toxicity, which led to discontinuations and dose reductions in almost half of the treated patients.

Despite the low dose intensity adjuvant chemotherapy was associated with a significantly improved relapse-free survival and a tendency towards an increased distant relapse-free survival in multivariate analysis.

The combination of dox containing adjuvant chemotherapy and postoperative radiation led to an increased incidence of radiation pneumonitis and tended to increase the incidence of hematological toxicity.

Tamoxifen adjuvant therapy when added to postoperative radiation and chemotherapy did not result in any overall improvement in local control, relapse free survival or overall survival. In the subset of estrogen receptor positive tumours, however, tamoxifen seemed to improve both relapse-free and overall survival, while there might even have been an adverse effect of tamoxifen in patients with estrogen receptor negative tumours.

The study has been supported by *Finska Läkaresällskapet* and Farmitalia Carlo Erbo, Scandinavia.

### References

ABELOFF, M.D., BEVERIDGE, R.A., DONEHOVER, R.C., FETTING, J.H., DAVIDSON, N.E., GORDON, G.G., WATERFIELD, W.C. & DAMRON, D.J. (1990). Sixteen-week dose-intensive chemotherapy in the adjuvant treatment of breast cancer. *JNCI*, **82**, 570–574.

BATIST, G. & ANDREWS, J.L. (1981). Pulmonary toxicity of antineoplastic cells. *JAMA*, **246**, 1449–1453.

BIANCO, A.R., DE PLACIDO, S., GALLO, C., PAGLIARULO, C., MARINELLI, A., PETRELLA, G., D'ISTRIA, M. & DELRIO, G. (1988). Adjuvant therapy with tamoxifen in operable breast cancer. 10 year results of the Naples (GUN) study. *Lancet*, **ii**, 1095–1099.

BRINCKER, H. (1988). Distant recurrence in breast cancer. Survival expectations and first choice of chemotherapy regimen. *Acta Oncol.*, **27**, 729–732.

BUDMAN, D.R., KORZUN, A.H., AINSER, J., YOUNGER, J., SILVER, R., COSTANZA, M., RICE, M.A. & WOOD, W. (1990). A feasibility study of intensive CAF as outpatient adjuvant therapy for stage II breast cancer in a cooperative group: CALGB 8443. *Cancer Invest.*, **8**, 571–575.

BUZDAR, A.U., KAU, S.W., SMITH, T.L. & HORTOBAGYI, G.N. (1989). Ten-year results of FAC adjuvant chemotherapy trial in breast cancer. *Am. J. Clin. Oncol.*, **12**, 123–128.

BUZDAR, A.U., MCNEESE, M.D., HORTOBAGYI, G.N., SMITH, T.L., KAU, S., FRASCHINI, G., HUG, V., ELLERBROEK, N., HOLMES, F.A., AMES, F. & SINGLETARY, E. (1990). Is chemotherapy effective in reducing the local failure rate in patients with operable breast cancer? *CAncer*, **65**, 394–399.

CASSADY, J.R., RICHTER, M.P., PIRO, A.J. & JAFFE, N. (1975). Radiation-adriamycin interactions: preliminary clinical observations. *Cancer*, **?**, 946-949.

CUZICK, J., STEWART, H., PETO, R., BAUM, M., FISHER, B., HOST, H., LYTHGOE, J.P., RIBEIRO, G., SCHEURLEN, H. & WALLGREN, A. (1987). Overview of randomized trials of postoperative adjuvant radiotherapy in breast cancer. *Cancer Treat. Rep.*, **71**, 15-29.

DOMBERNOWSKY, P., BRINCKER, H., HANSEN, M., MOURIDSEN, H.T., OVERGAARD, M., PANDURO, J., ROSE, C., AXELSSON, C.K., ANDERSEN, J. & ANDERSEN, K.W. (1988). Adjuvant therapy of premenopausal and menopausal high-risk breast cancer patients. Present status of the Danish Breast Cancer Cooperative Groups Trials 77-B and 82-B. *Acta Oncol.*, **27**, 691-697.

EBCTCG (1988). Effects of adjuvant tamoxifen and of cytotoxic therapy on mortality in early breast cancer. An overview of 61 randomized trials of 28,896 women. *N. Engl. J. Med.*, **319**, 1681-1692.

EBCTCG (1990). *Treatment of Early Breast Cancer. Worldwide Evidence 1985-1990*. Oxford University Press: Oxford.

EBCTCG (1992). Systemic treatment of early breast cancer by hormonal, cytotoxic, or immune therapy. 133 randomised trials involving 31,000 recurrences and 24,000 deaths among 75,000 women. Early Breast Cancer Trialists' Collaborative Group. *Lancet*, **339**, 1-15, 71-85.

FISHER, B., BROWN, A.M., DIMITROV, N.V., POISSON, R., REDMOND, C., MARGOLESE, R.G., BOWMAN, D., WOLMARK, N., WICKERHAM, D.L., KARDINAL, C.G., SHIBATA, H., PATERSON, A.H.G., SUTHERLAND, C.M., ROBERT, N.J., AGER, P.J., LEVY, L., WOLTER, J., WOZNIAK, T., FISHER, E.R. & DEUTSCH, M. (1990). Two months of doxorubicin-cyclophosphamide with and without interval reinduction therapy compared with 6 months of cyclophosphamide, methotrexate, and fluorouracil in positive-node breast cancer patients with tamoxifen-nonresponsive tumors: results from the National Surgical Adjuvant Breast and Bowel Project B-15. *J. Clin. Oncol.*, **8**, 1483-1496.

FISHER, B., REDMOND, C., BROWN, A., FISHER, E.R., WOLMARK, N., BOWMAN, D., PLOTKIN, D., WOLTER, J., BORNSTEIN, R., LEGAULT-POISSON, S. & SAFFER, E.A. (1986). Adjuvant chemotherapy with and without tamoxifen in the treatment of primary breast cancer: 5-year results from the National Surgical Adjuvant Breast and Bowel Project trial. *J. Clin. Oncol.*, **4**, 459-471.

FISHER, B., REDMOND, C., BROWN, A., WOLMARK, N., WITTLIFF, J., FISHER, E.R., PLOTKIN, D., BOWMAN, D., SACHS, S., WOLTER, J., FRELICK, R., DESSER, R., LICALZI, N., GEGGIE, P., CAMPBELL, T., ELIAS, E.G., PRAGER, D., KOONTZ, P., VOLK, H., DIMITROV, N., GARDNER, B., LERNER, H. & SHIBATA, H. (1981). Treatment of primary breast cancer with chemotherapy and tamoxifen. *N. Engl. J. Med.*, **305**, 1-6.

FISHER, B., REDMOND, C., WICKERHAM, D.L., BOWMAN, D., SCHIPPER, H., WOLMARK, N., SASS, R., FISHER, E.R., JOCHIMSEN, P., LEGAULT-POISSON, S., DIMITROV, N., WOLTER, J., BORNSTEIN, R., ELIAS, E.G., LICALZI, N., PATERSON, A.H.G. & SUTHERLAND, C.M. (1989). Doxorubicin-containing regimens for the treatment of stage II breast cancer: The National Surgical Adjuvant Breast and Bowel Project experience. *J. Clin. Oncol.*, **7**, 572-582.

GINSBERG, S.J. & COMIS, R.L. (1982). The pulmonary toxicity of antineoplastic agents. *Semin. Oncol.*, **9**, 34-51.

GRÖHN, P., HEINONEN, E., KLEFSTRÖM, P. & TARKKANEN, J. (1984). Adjuvant postoperative radiotherapy, chemotherapy and immunotherapy in stage III breast cancer. *Cancer*, **54**, 670-674.

HØST, H. & BRENNHOVD, I.O. (1977). The effect of post-operative radiotherapy in breast cancer. *Int. J. Radiation Oncol. Biol. Phys.*, **2**, 1061-1067.

MARSHALL, J.S., GORDON, N.H., HUBAY, C.A. & PEARSON, O.H. (1987). Assessment of tamoxifen as adjuvant therapy in stage II breast cancer: a long-term follow-up. *J. Lab. Clin. Med.*, **109**, 300-307.

MAURIAC, L., DURAND, M., CHAUVERGNE, J., BONICHON, F., AVRIL, A., MAGE, P., DILHUYDY, M.H., LETREUT, A., WAFFLART, J., MAREE, D. & LAGARDE, C. (1988). Adjuvant trial for stage II receptor positive breast cancer: CMF vs. CMF+tamoxifen in a single centre. *Breast Cancer Res. & Treat.*, **11**, 179-186.

MAYER, E.G., POULTER, C.A. & ARISTIZABAL, S.A. (1976). Complications of irradiation related to apparent drug potentiation by adriamycin. *Int. J. Radiat. Oncol. Biol. Phys.*, **1**, 1179-1188.

MORRISON, J.M., HOWELL, A., KELLY, K.A., GRIEVE, R.J., MONYPENNY, I.J., WALKER, R.A. & WATERHOUSE, J.A. (1989). West Midlands Oncology Association trials of adjuvant chemotherapy in operable breast cancer: results after a median follow-up of 7 years. I. Patients with involved axillary lymph nodes. *Br. J. Cancer*, **60**, 911-918.

MOURIDSEN, H. & OVERGAARD, M. (1990). *The Role of Adjuvant Radiotherapy Added to Systemic Therapy*. Proceedings of 15th International Cancer Congress. Hamburg, BRD, Springer International.

MOURIDSEN, H.T., ROSE, C., OVERGAARD, M., DOMBERNOWSKY, P., PANDURO, J., THORPE, S., RASMUSSEN, B.B., BLICHERT, T.M. & ANDERSEN, K.W. (1988). Adjuvant treatment of postmenopausal patients with high risk primary breast cancer. Results from the Danish adjuvant trials DBCG 77 C and DBCG 82 C. *Acta Oncol.*, **27**, 699-705.

OHNOSHI, T., HIRAKI, S., KAWAHARA, S., YAMASHITA, H., YONEI, T., ISHII, J., EGAWA, T., KOZUKA, A., HIRAKI, Y. & KIMURA, I. (1986). Randomized trial comparing chemotherapy alone and chemotherapy plus chest irradiation in limited stage small cell lung cancer: a preliminary report. *Jpn. J. Clin. Oncol.*, **16**, 271-277.

PATEL, A.R., SHAH, P.C., RHEE, H.L., SASSOON, H. & RAO, K.P. (1976). Cyclophosphamide therapy and interstitial fibrosis. *Cancer*, **38**, 1542-1549.

PERLOFF, M., NORTON, L., KORZUN, A., WOOD, W., CAREY, R., WEINBERG, V. & HOLLAND, J.F. (1986). Advantage of and Adriamycin (A) combination plus Halotestin (H) after initial cyclophosphamide, 5-fluorouracil, vincristine and prednison (CMFVP) for adjuvant treatment of node positive stage II breast cancer. *Proc. Am. Soc. Clin. Oncol.*, **5**, 70 (abstr.).

ROSIELLO, R.A. & MERRILL, W.W. (1990). Radiation-induced lung injury. *Clin. Chest. Med.*, **11**, 65-71.

RUTQVIST, L.E., CEDERMARK, B., GLAS, U., JOHANSSON, H., ROTSTEIN, S., SKOOG, L., SOMELL, A., THEVE, T., WILKING, N., ASKERGREN, J. & HJALMAR, M.L. (1990). Randomized trial of adjuvant tamoxifen combined with postoperative radiation therapy or adjuvant chemotherapy in postmenopausal breast cancer. *Cancer*, **66**, 89-96.

SENANAYAKE, F. (1984). Adjuvant hormonal chemotherapy in breast cancer: early results from a controlled trial. *Lancet*, **i**, 1148-1149.

SULKES, A., BRUFMAN, G., RIZEL, S., WESHLER, Z., BIRAN, S. & FUKS, Z. (1983). The effect of postoperative radiotherapy on the feasibility of optimal dose adjuvant CMF chemotherapy in stage II breast cancer. *In. J. Radiat. Oncol. Biol. Phys.*, **9**, 17-21.

TAYLOR, S.G., KNUIMAN, M.W., SLEEPER, L.A., OLSON, J.E., TORMEY, D.C., GILCHRIST, K.W., FALKSON, G., ROSENTHAL, S.N., CARBONE, P.P. & CUMMINGS, F.J. (1989). Six-year results of the Eastern Cooperative Oncology Group Trial of observation versus CMFP versus CMFPT in postmenopausal patients with node-positive breast cancer. *J. Clin. Oncol.*, **7**, 879-889.

TORMEY, D.C., GRAY, R., GILCHRIST, K., GRAGE, T., CARBONE, P.P., WOLTER, J., WOLL, J.E. & CUMMINGS, F.J. (1990). Adjuvant chemohormonal therapy with cyclophosphamide, methotrexate, 5-fluorouracil, and prednison (CMFP) or CMFP plus tamoxifen compared with CMF for premenopausal breast cancer patients. An Eastern Cooperative Oncology Group trial. *Cancer*, **65**, 200-206.

VERSCHOORE, J., LAGRANGE, J.L., BOUBLIL, J.L., AUBANEL, J.M., BLAIVE, B., PINTO, J. & NAMER, M. (1987). Pulmonary toxicity of a combination of low-dose doxorubicin and irradiation for inoperable lung cancer. *Radiother. Oncol.*, **9**, 281-288.

WENDT, A.G., JONES, S.E., SALMON, S.E., GIORDANO, G.F., JACKSON, R.A., MILLER, R.S. & HEUSINKVELD, R.S. (1979). Adjuvant treatment of breast cancer with adriamycin-cyclophosphamide with or without radiation therapy. In *Adjuvant Therapy of Cancer II*, Jones, S.E. & Salmon, S.E. (eds), pp. 285-293. Grune & Stratton: New York.

WHITE, D.A., ORENSTEIN, M., GODWIN, A. & STOVER, D.E. (1984). Chemotherapy-associated pulmonary toxic reactions during treatment for breast cancer. *Arch. Intern. Med.*, **144**, 953-956.